# Quantitative Multispectral Imaging Differentiates Melanoma from Seborrheic Keratosis

**DOI:** 10.3390/diagnostics11081315

**Published:** 2021-07-22

**Authors:** Szabolcs Bozsányi, Klára Farkas, András Bánvölgyi, Kende Lőrincz, Luca Fésűs, Pálma Anker, Sára Zakariás, Antal Jobbágy, Ilze Lihacova, Alexey Lihachev, Marta Lange, Dmitrijs Bliznuks, Márta Medvecz, Norbert Kiss, Norbert M. Wikonkál

**Affiliations:** 1 Department of Dermatology, Venereology and Dermatooncology, Semmelweis University, 1085 Budapest, Hungary; bozsanyi.szabolcs@med.semmelweis-univ.hu (S.B.); farkas.klara@phd.semmelweis.hu (K.F.); banvolgyi.andras@med.semmelweis-univ.hu (A.B.); lorincz.kende@med.semmelweis-univ.hu (K.L.); fesus.luca@med.semmelweis-univ.hu (L.F.); anker.palma@phd.semmelweis.hu (P.A.); zakarias.sara@phd.semmelweis.hu (S.Z.); jobbagy.antal@med.semmelweis-univ.hu (A.J.); medvecz.marta@med.semmelweis-univ.hu (M.M.); wikonkal.norbert@med.semmelweis-univ.hu (N.M.W.); 2 Biophotonics Laboratory, Institute of Atomic Physics and Spectroscopy, University of Latvia, LV-1004 Riga, Latvia; ilze.lihacova@lu.lv (I.L.); aleksejs.lihacovs@lu.lv (A.L.); marta.lange@lu.lv (M.L.); 3 Faculty of Computer Science and Information Technology, Riga Technical University, LV-1048 Riga, Latvia; dmitrijs.bliznuks@rtu.lv

**Keywords:** melanoma, seborrheic keratosis, autofluorescence imaging, LED, dermoscopy, quantitative analysis, diffuse reflectance imaging, diagnosis

## Abstract

Melanoma is a melanocytic tumor that is responsible for the most skin cancer-related deaths. By contrast, seborrheic keratosis (SK) is a very common benign lesion with a clinical picture that may resemble melanoma. We used a multispectral imaging device to distinguish these two entities, with the use of autofluorescence imaging with 405 nm and diffuse reflectance imaging with 525 and 660 narrow-band LED illumination. We analyzed intensity descriptors of the acquired images. These included ratios of intensity values of different channels, standard deviation and minimum/maximum values of intensity of the lesions. The pattern of the lesions was also assessed with the use of particle analysis. We found significantly higher intensity values in SKs compared with melanoma, especially with the use of the autofluorescence channel. Moreover, we found a significantly higher number of particles with high fluorescence in SKs. We created a parameter, the SK index, using these values to differentiate melanoma from SK with a sensitivity of 91.9% and specificity of 57.0%. In conclusion, this imaging technique is potentially applicable to distinguish melanoma from SK based on the analysis of various quantitative parameters. For this application, multispectral imaging could be used as a screening tool by general physicians and non-experts in the everyday practice.

## 1. Introduction

Melanoma (malignant melanoma, MM) is a melanocytic tumor that is responsible for most skin cancer-related deaths [1,2]. Worldwide, about 232,100 new patients are diagnosed with melanoma annually, and it accounts for about 55,000 deaths every year [3]. It has four main subtypes that include superficial spreading melanoma (SSM), nodular melanoma (NM), lentigo maligna melanoma (LMM) and acral lentiginous melanoma (ALM). Other than these, rare variants, such as amelanotic, desmoplastic melanoma or uveal melanoma also exist [2,4]. Unlike most other types of cancer, melanoma bears with an extreme metastatic potential and hence mortality when compared to the total tumor burden. For this reason, early diagnosis is crucial for the patients’ long-term survival. The visibility and easy accessibility of melanoma creates an opportunity for various imaging methods and screening devices. Early diagnosis is the most important factor in the successful management of melanoma [5,6], where dermoscopy plays a pivotal role. Dermoscopes are epiluminescence microscopes that typically use a 10× magnification to unveil the more detailed structure of the lesion [7,8]. The most widely applied dermoscopic algorithm for pigmented skin lesions is the ‘Chaos and clues’ revised pattern analysis. ‘Chaos’ refers to the presence of dermoscopic asymmetry of the pattern, color and border abruptness. If chaos is present, we examine the lesion for the nine possible clues for malignancy. The clue patterns include the grey or blue structures, eccentric structureless area, thick lines (reticular or branched), peripheral black dots or clods, radial lines or pseudopods, white lines, polymorphous vessels, parallel lines/ridges, or polygons. There are also certain exceptions to improve diagnostic sensitivity. Intervention is needed if the lesion is changing on an adult, it is nodular and/or comprises small lesions with any clue to malignancy, lesion on the head or neck, with pigmented circles and/or dermoscopic grey or volar lesion with parallel ridge pattern. If a clue is present, excision or biopsy is needed unless an unambiguous diagnosis of seborrheic keratosis (SK) can be made by pattern analysis [9].

SK is a common benign epithelial skin tumor. Clinically, early lesions evolve most often from solar lentigines, which are oval, light-to-dark-brown macules, with sharply demarcated borders. Advanced lesions transform into plaques and have a typical stuck-on appearance [10]. They are mostly hyperkeratotic and acanthotic, and their melanisation is common [11]. The dermoscopic criteria for SK according to the ‘Chaos and clues’ pattern analysis are the presence of multiple orange clod, multiple white clods, sharply demarcated border over total periphery and the pattern of thick, curved lines [9]. Histologically, these findings are equivalent to the papillomatous epidermis, pseudo-horn or milia-like cysts, enlarged dermal capillaries and intraepidermal cysts [12,13]. Comedo-like openings are keratin-filled invaginations of the epidermis. Milia-like cysts are intraepidermal keratin pseudocysts with a whitish-yellow appearance under non-polarized dermoscopy [12,13]. Moth-eaten border is an additional characteristic feature, which means that the lesion is sharply demarcated with small waves along the edges [14].

SKs develop most commonly among elderly patients and in large numbers [15,16,17]. In certain cases, when a younger patient is affected, and the number of simultaneous lesions is low, diagnostic challenges may more likely occur. The clinical diagnosis of SK is most often made without histological confirmation, while skin biopsy or diagnostic excision is reserved for ambiguous cases [15,18]. As a benign condition, SK usually does not need any further treatment, or may be removed using cryosurgery, curettage, electrocautery or laser ablation [17].

**Figure 1:**
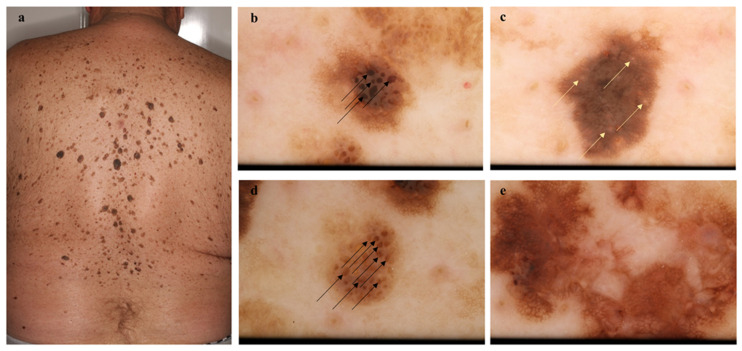
Melanoma among several seborrheic keratoses (SK) on the back. This 67-year-old male patient was diagnosed with a melanoma on his back (in situ melanoma, pTis) near the shoulders and many SK lesions (**a**). Non-polarized dermoscopic images of SKs (**b**–**d**) and melanoma (**e**). SKs (**b**–**d**) show a dull surface, including fingerprint and cerebriform patterns, milia-like cyst (yellow arrows) and comedo-like opening (black arrows). Melanoma (**e**) contains an irregular pigment network and blue-white veil with multiple colors. The case of this patient also emphasizes the importance of full body examinations.

The differential diagnosis of melanoma and SK is crucial, as occasionally they can have very similar clinical morphology and may also mimic each other [19]. It is especially hard to differentiate nodular melanoma from verrucous SKs because the surface of melanomas can be also verrucous [20,21,22] or scaly, it can be hyperkeratotic, and it can have epidermal hyperplasia, milia-like cysts or even comedo-like openings, especially in case of folliculotropism [23]. For the differentiation of SK and melanoma, dermoscopes are relatively cheap and widely available tools in the routine clinical practice [14,24]. However, they require special training and expertise, and not widely used among general physicians. Full body examination using dermoscopy is time consuming, and it may prove to be difficult to detect melanoma among a high number of SKs (Figure 1) [25,26]. There are two main modes of dermoscopy, polarized and non-polarized. Non-polarized dermoscopy is optimal for the evaluation of seborrheic keratosis as it visualizes milia-like cysts and comedo-like openings more clearly [27]. In addition, there are various further imaging modalities for the diagnosis of skin tumors, such as high-frequency ultrasound [28,29], optical coherence tomography [30], reflectance confocal microscopy (RCM) [31] and multiphoton microscopy [32,33,34]. However, these are very expensive and their availability is generally limited to large dermatology centers [35,36]. There is a subgroup of melanomas that appear very similar to SKs and, as new entities, are recently referred to as SK-like melanomas [18]. They cannot be differentiated from SKs with the naked eye or are not even discernible with a dermoscope [37]. Among SKs, there is also a subgroup, the MM-like SKs, which can only be discriminated using dermoscope. A recent study by Farnetani et al. used RCM to diagnose challenging SKs of the face. In this work, RCM proved to be a suitable diagnostic tool to prevent unnecessary excisions of such lesions [38].

Multispectral imaging (MSI) is a promising tool for in vivo skin cancer detection. MSI utilizes different wavelength bands to acquire images of the skin [36], mostly the visible and the infrared spectrum of the light (400–970 nm) where the light source is usually provided by halogen lamps or LEDs [39]. During MSI, a set of images is taken from the same skin location with the use of different wavelength bands [40]. Compared with other imaging modalities, MSI is a cost-effective technique, which can be implemented also to smartphone cameras that render this technique easily accessible [41,42]. The use of MSI in the field of dermatology has been emerging in the last two decades. An LED-based MSI device was recently successfully introduced for the detection of recurring skin cancers [43,44] and proved to be applicable in the differentiation of different tumor types from benign lesions based on their mean intensity of autofluorescence (AF) [45]. In addition, MSI has been also utilized for the detection of rare skin disorders [46,47].

The aim of the present study was to investigate spectral reflectance and autofluorescence properties of melanoma and SK to assess their usefulness for the accurate differentiation of these two disorders.

## 2. Materials and Methods

### 2.1. Patient Data

MSI was performed at the Department of Dermatology, Venereology and Dermatooncology, Semmelweis University (Budapest, Hungary) and at the Oncology Centre of Latvia (Riga, Latvia). We have examined a total of 266 patients with melanoma or SK and took one or more image sets of their lesions depending on the lesions’ number and size. Many patients with SKs had several lesions increasing the number of image sets taken. A total of 127 patients (161 image sets) had histologically proven melanomas from which 66 were SSM (52%), 18 NMs (14.1%), 21 in situ melanomas (16.5%), 3 ALM (2.3%), 1 LMM (1%) and 18 unclassified (14.1%). Six patients had SK resembling MMs (6 image sets). Comparison was made to 139 patients (319 SK lesions with 319 image sets) diagnosed with SK by dermatologists (2–3 SKs/patient) with the use of a commercial Heine Delta 20 (HEINE Optotechnik GmbH & Co. KG, Gilching, Germany) dermoscope. Thirty patients had MM resembling SKs (52 image sets). The mean age of patients with melanoma and SK was 64.09 ± 13.55 and 70.19 ± 11.147 years, respectively. The gender ratio was 44% women and 56% men, among patients with melanoma, and 44.9% women and 55.1% men, among patients with SK. This study was approved by the Ethics Committee of Semmelweis University (SE RKEB no. 228/2018) and by the Research Ethics Committee of Institute of Cardiology and Regenerative Medicine, University of Latvia (approved on: 26 February 2019).

### 2.2. Multispectral Imaging

The handheld prototype used in this study was developed by the University of Latvia in collaboration with Riga Technical University (Riga, Latvia). The illumination source is an LED-ring which contains four types of LEDs (SML-LXL8047UVC, Lumex, Inc., Ronkonkoma, NY, USA) with wavelengths of 405 nm to induce skin autofluorescence (AF) and 525 nm green (G), 660 nm red (R) and 940 nm infrared (IR) [48,49], from which we used AF, G and R images for our further analyses. The lights penetrate to different layers of the skin with irradiating power density of 20 mW/cm^2^. Images were collected with a color CMOS 5 megapixel IDS camera (MT9P006STC, IDS uEye UI3581LE-C-HQ, Obersulm, Germany) fixed at 60 mm distance from the illuminated skin with a field of view of 2 × 2 cm^2^ [35]. A long pass filter (T515 nm > 90%) was inserted in front of the camera to block 405 nm excitation illumination. AF was captured in G and R spectral channels. In cases where the lesion surface was not flat (e.g., fingers, elbows and so on) the image focus was slightly adapted by the adjustment of the region of interest (ROI). The acquired images were automatically transferred to a cloud server, as described earlier [50]. The description of this prototype device can be found in [35,51]. To avoid the decrease in the induced AF intensity caused by photobleaching process [35], all images were taken during the first second of LED exposure. A black marker was applied next to the lesions to improve image alignment (area: 0.125 cm^2^).

### 2.3. Quantitative Intensity Descriptors

Image analysis was performed with ImageJ v1.46 software (NIH, Bethesda, MD, USA) [52]. For the intensity analysis, we manually selected the skin lesions ROI using the AF, G and R channels. We analyzed the intensity including minimum and maximum, mean intensity value and standard deviation (SD), to compare melanomas with SKs regarding these parameters. We calculated ratios of the intensity values of the different channels including AF, G and R and used these ratios (AF/G, AF/R) to differentiate melanomas from SKs. We also measured the ratio of the pixels with the lowest and highest intensity values within each lesion (Min/Max). Mean AF intensities of the healthy skin were calculated using the intensity values of three additional skin areas (75 × 75 pixels) with uniform distribution of intensity values selected manually outside the lesions. For normalization, we divided the intensity values of the lesions to that of the adjacent control skin.

### 2.4. Analyis of Particles with High Fluorescence

Particle analysis was conducted to count and analyze the particles with high fluorescence values. We developed an algorithm within the ImageJ framework to analyze the images. AF Images were converted to 8-bit to be suitable for automated thresholding. Automated default thresholding of the ImageJ software was used. After this step, a particle analysis was performed, where the size of the particles was specified between the range of 10 and 100,000 pixel^2^, the range of circularity was set between 0.4 and 1.0, with the edges excluded. If there were two or more SK lesions in one field of view, a ROI was selected manually to specify the lesion for the particle analysis. ROI was also selected manually in lesions of the hairy scalp where hair affected the correct measurement. The area percentage was calculated from the ratio of the area of the particles with high fluorescence values/area of the lesion.

### 2.5. Calculating the SK Index

We used those parameters, which showed a significant difference between SKs and melanomas to create Equation (1) to calculate the SK index. We set the threshold of SK index to 30 (arbitrary unit), classifying the lesions below 30 as melanomas and classifying the lesions over 30 as SKs.

(1) $$\mathrm{SK}\;\mathrm{index}=\frac{2\cdot \mathrm{AF}\cdot \mathrm{StDev}\cdot \left(\frac{\mathrm{Min}}{\mathrm{Max}}\right)}{G\cdot R}+\left(\mathrm{Particle}\;\mathrm{number}\cdot \mathrm{Area}\;\%\right)$$

### 2.6. Statistical Analysis

Welch’s *t*-tests were used for statistical analysis, as applicable. We used receiver operating characteristic (ROC) curves to count the area under the curves (AUC) with default settings (Wilson/Brown method with a confidence interval of 95%). Statistical tests were performed with the use of GraphPad Prism v8.0.1. software (GraphPad Software Inc., La Jolla, CA, USA). We used contingency tables to calculate sensitivity and specificity based on the SK indexes. *p* values below 0.05 were considered statistically significant. The results are expressed as mean ± standard error.

### 2.7. Inclusion Criteria

In this study, we included melanomas histologically verified by dermatopathologists and SKs confirmed by two expert dermatologists with clinical and dermoscopic examination. Only lesions on body parts accessible to the MSI device were selected. If the lesion was larger than the field of view, more image sets were taken.

### 2.8. Exclusion Criteria

Clinically diagnosed melanomas that have not been validated histologically were also excluded. In addition, ulcerated lesions were excluded from the particle analysis.

## 3. Results

### 3.1. Intensity Values

**Figure 2:**
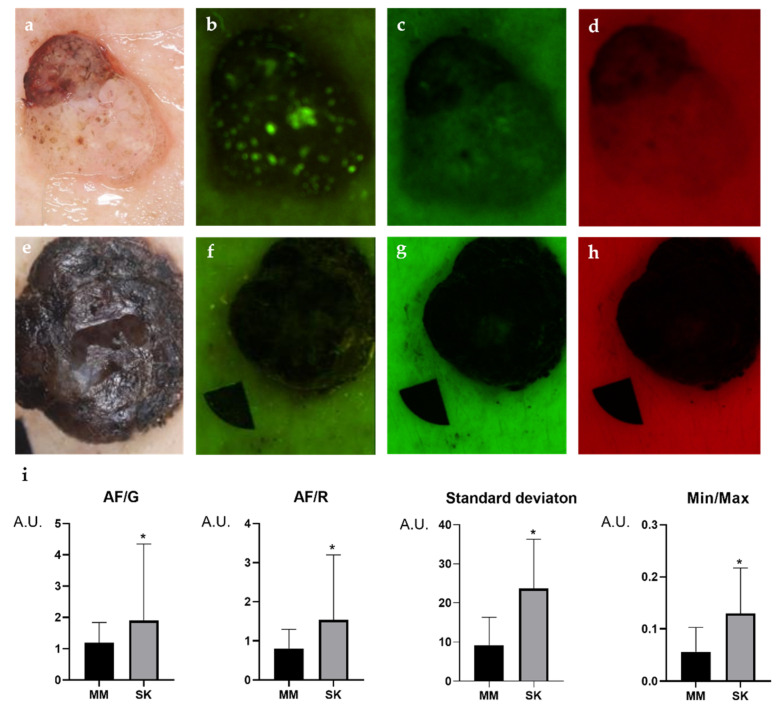
Multispectral LED images comparing SK (**a**–**d**) to malignant melanoma (**e**–**h**), and the results of statistical analysis of intensity parameters (**i**). Dermoscopic images of the lesions (**a**,**e**), 405 nm autofluorescence (AF) channel (**b**,**f**), 525 nm green (G) (**c**,**g**) 660 nm red (R) channel images (**d**,**h**). Welch’s *t*-test was used to compare the intensity values of the lesions. In SKs, all the intensity values were significantly higher compared with melanomas (**i**). The AF/G and AF/R ratios were normalized to adjacent control skin. A.U., arbitrary unit. *p* values below 0.0001 were considered statistically significant. Means ± SD: AF/G: 1.187 ± 0.647 (MM) vs. 1.891 ± 2.437 (SK), AF/R: 0.809 ± 0.48 (MM) vs. 1.53 ± 0.48 (SK), Standard deviation: 9.23 ± 7.1 (MM) vs. 23.6 ± 12.58 (SK), Min/Max: 0.056 ± 0.045 (MM) vs. 0.129 ± 0.087 (SK). * *p* < 0.05.

**Figure 3:**
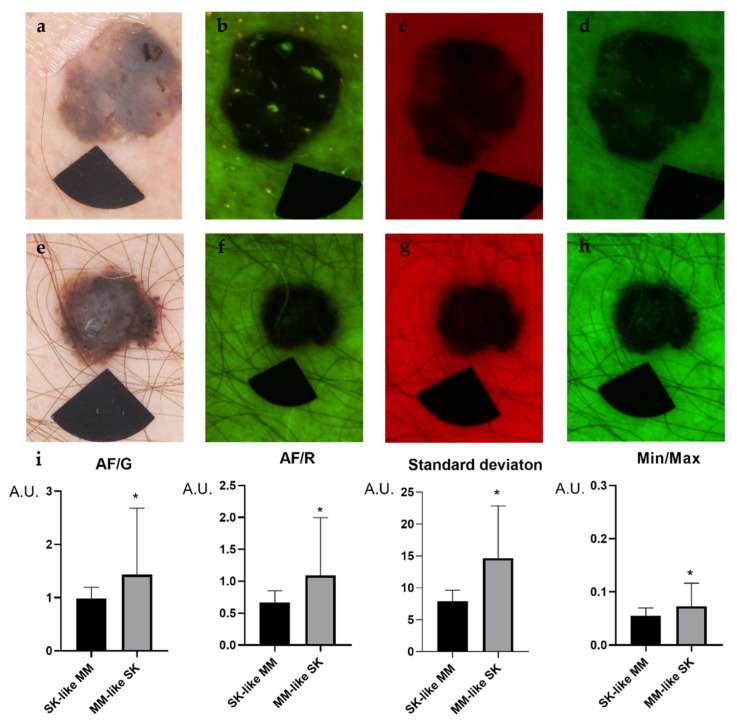
Representative images of clinically challenging melanoma (MM)-like SKs and SK-like MMs. The upper row is an SK-mimicking melanoma (MM) (nodular melanoma, Breslow:1.84, Clark III., pT2a) while the lower row is a melanoma-resembling SK. Dermoscopy images of the lesions (**a**,**f**), 405 nm autofluorescence (AF) channel (**b**,**g**), 660 nm red (R) channel images (**c**,**h**) 525 nm green (G) (**d**,**i**) and the results of particle analysis (**e**,**j**). Welch’s *t*-test was used to compare the intensity values of the lesions. In SKs, all of the intensity values were significantly higher compared with melanomas (**i**). A.U., arbitrary unit. *p* values below 0.05 were considered statistically significant. Means ± SD: AF/G: 0.984 ± 0.212 (SK like MM) vs. 1.415 ± 1.215 (MM like SK), AF/R: 0.667 ± 0.183 (SK like MM) vs. 1.08 ± 0.884 (MM like SK), Standard deviation: 7.9 ± 1.745 (SK like MM) vs. 14.79 ± 7.96 (MM like SK), Min/Max: 0.05 ± 0.01 (SK like MM) vs. 0.073 ± 0.043 (MM like SK). * *p* < 0.05.

In SK, both the AF/G, AF/R and Min/Max ratios proved to be significantly higher compared with the melanomas (Figure 2 and Figure 3). Milia-like cysts and comedo-like openings showed very high AF intensities, which appeared as small and dense signals inside the lesion leading to AF disparities (Figure 2 and Figure 3). Disproportions in intensity values of the SK lesions resulted in significantly higher SD compared with melanomas. While milia-like cysts and comedo like openings appeared as bright particles embedded into SK lesions, melanomas did not contain such particles (Figure 2 and Figure 3).

### 3.2. Particle Analysis

**Figure 4:**
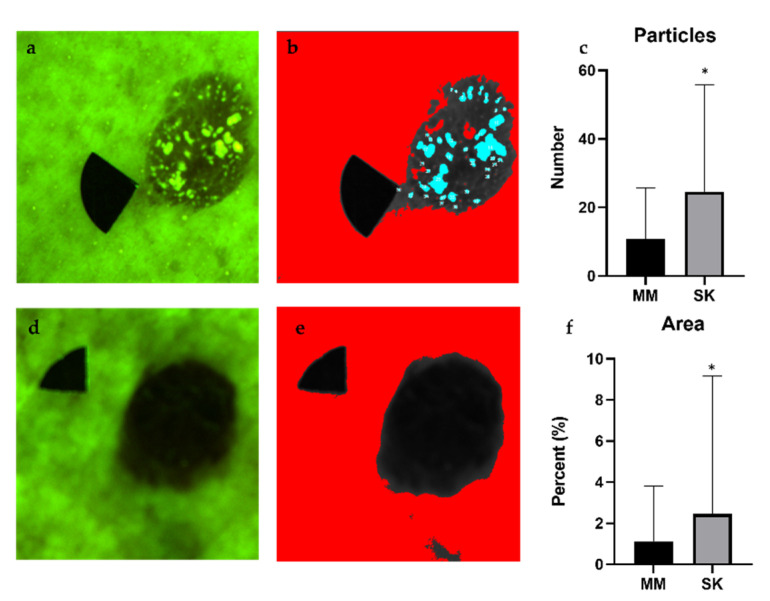
Particle analysis of SKs (**a**,**b**) and melanomas (**d**,**e**) and the results of statistical analysis, including the number of particles with high fluorescence values (**c**) and the total area of these particles (**f**). We used the Overlay Masks option of the ImageJ software to visualize the particles (shown in blue color) in these images. Comparing the melanoma values to SK values using Welch’s *t*-test, SKs contained a significantly higher number of particles, which were also significantly larger (**c**). The percentage of the area covered by the particles was significantly smaller in melanomas compared with SKs (**f**). Here, the limitations of the algorithm were also visible, because, with these settings, not all of the particles could be selected and measured (white dots on panel (**b**)). *p* values below 0.0001 were considered statistically significant. Means ± SD: Particles: 10.76 ± 14.9 (MM) vs. 23.56 ± 30.44 (SK), Area: 1.126 ± 2.698% (MM) vs. 6.412 ± 13.26% (SK). * *p* < 0.05.

**Figure 5:**
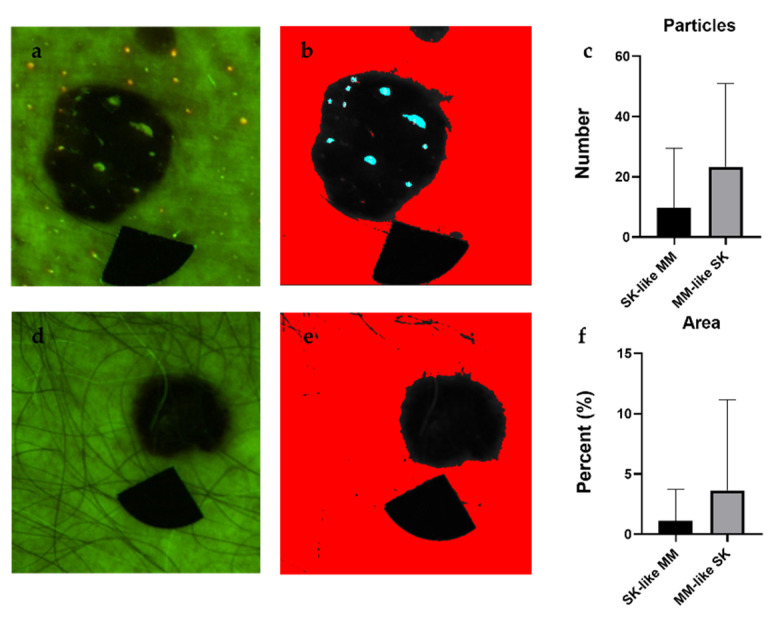
Particle analysis of melanoma (MM)-like SKs (**a**,**b**) and SK-like MMs (**d**,**e**) and the results of statistical analysis, including the number of particles with high fluorescence values (**c**) and the total area of these particles (**f**). We used the Overlay Masks option of the ImageJ software to visualize the particles (shown in blue color) in these images. The differences of the number of the particles detected and their area % of the lesions were not significantly different between the two groups using Welch’s *t*-test. *p* values below 0.05 were considered statistically significant. Means ± SD: Particles: 9.667 ± 19.87 (SK like MM) vs. 22.42 ± 27.10 (MM like SK), Area: 1.11 ± 2.60% (SK like MM) vs. 3.56 ± 7.31% (MM like SK).

After particle analysis, melanomas appeared as lesions with uniform intensity, whereas SKs had many high intensity particles embedded in the lesions (Figure 4 and Figure 5). The algorithm counted a significantly higher number of particles in SKs, which took a greater part (Area%) of the lesion (Figure 4 and Figure 5). The percentage of melanomas excluded from the analysis amounted to no more than 5% of the total number of lesions.

### 3.3. SK Index

**Figure 6:**
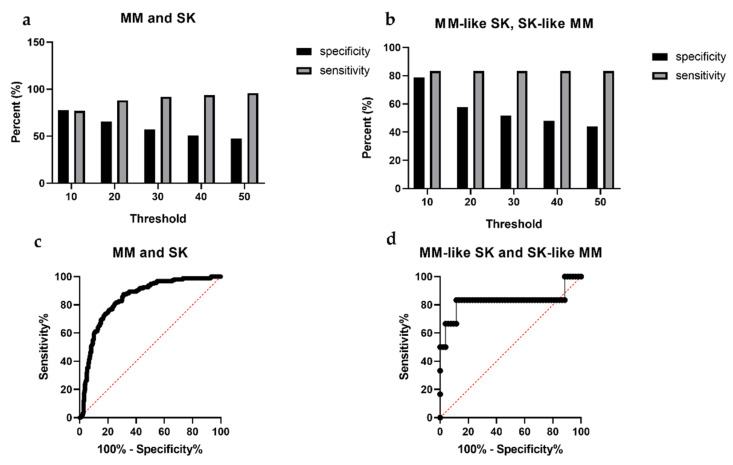
Sensitivity and specificity values using different SK index threshold parameters and receiver operating characteristic (ROC) curves for melanoma prediction. Melanoma (MM) group compared to SK group (**a**) and MM-like SK group compared to SK-like MM (**b**). We used different thresholds of the SK index and 30 proved to be the most suitable for a screening device, where the sensitivity is 91.9% (over 90%) and specificity is 57.0% (over 50%). Setting the right threshold was important from the aspect of the sensitivity and specificity for a potential screening tool. The 30 threshold was suitable with a sensitivity of 83.3% and specificity of 51.9% for a potential screening device. ROC was performed to show the differences between the comparison of the MM and SK (**c**) and MM-like SK and SK-like MM groups (**d**). The Area Under the Curve (AUC) was 0.844 (patients: MM, control: SK, 95% confidence interval, *p* < 0,0001) in the MM and SK ROC curve (**c**). AUC was 0.826 (patients: SK-like MM, control: MM-like SK, 95% confidence interval, *p* = 0.0092) in ROC curve of the MM-like SK and SK-like MM comparison. Y-axis: sensitivity, x-axis: 1-specificity. Red diagonal dotted line represents a non-discriminatory test (**c**,**d**).

The sensitivity of the SK index proved to be 91.9%, with a specificity of 57.0%. The positive predictive value was 51.7%, while the negative predictive value was 93.3%. Among the melanoma-like SKs and SK-like melanomas, the sensitivity was 83.3%, while the specificity was 51.9%. The positive predictive value was 16.6% and the negative predictive value was 92.8% (Figure 6). The ROC AUC analysis also showed significant differences. The comparison of MM and SK groups showed an AUC of 0.844, *p* < 0.0001 (patient group: MM, control: SK, 95% confidence interval) while the comparison of MM-like SK and SK-like MM groups showed an AUC of 0.826, *p* = 0.0092 (patient: SK-like MM, control: MM-like SK, 95% confidence interval) (Figure 6).

## 4. Discussion

The diagnosis of malignant melanoma is challenging in a significant number of patients. Even expert dermatologists may miss the diagnosis of melanoma in 16% of the cases [22], and it is even more frequently on unusual skin sites, such as the foot [53]. Delayed diagnosis of melanoma leads to dramatically lower life expectancy [54] and higher risk of metastatic disease [55]. There are many skin disorders which may resemble melanoma, including dysplastic nevus, lentigo maligna, congenital and acquired pigmented nevi, non-melanoma skin cancers, Bowen’s disease, actinic keratosis, Spitz-nevus, blue nevus, hemorrhage and SK [14,19,56,57,58,59,60]. There is an increasing number of multispectral imaging techniques in the market developed to diagnose skin diseases since 1994 [61,62]. Many of these techniques became commercialized, such as SIAscope [63], MelaFind [59] and SpectroShade [64] to aid the diagnosis of melanoma. However, their high price and insufficient specificity limit their use in the everyday clinical practice [65,66].

In this study, we compared melanoma to SK with an LED-based technique, using multiple quantitative parameters. We found that intensity parameters are capable of the differentiation between these two entities. In the AF images of SK, based on the comparison with dermoscopy images, high intensity values were mainly caused by the milia-like cysts and comedo-like openings, which are primarily filled with keratin [67]. However, keratin is not solely responsible for the high intensity values. NADH, FAD, complex structures and lipid particles may also be responsible for higher AF signal [68,69]. Compared with SK, melanoma images had lower AF intensity values, in agreement with the data in the literature [48,49]. The presence of melanin, which has a very specific absorption pattern, could be the explanation for the decreased AF signal, [48], but the altered collagen structure caused by the tumor growth may also play a part. [70,71]. Melanin acts as a nonfluorescent pigment under UV and short wavelength visible light; it only bears with fluorescent characteristics under near-infrared light [72]. The latter was confirmed with our measurements, where melanin appeared as a black nonfluorescent pigment visualized with AF, G and R channels without fluorescent emission, thus characterized by low intensity values. Both AF/G and AF/R ratios were significantly higher in SK, which is mainly caused by the high values in the AF channel. The average AF intensity of the SK lesion was also significantly higher compared with melanomas, which was in line with the Min/Max ratios, where the minimum values were notably higher in SKs.

The results of the particle analysis were more remarkable, but the standard deviation was high. There were also some difficulties where hyperkeratosis or ulcerations on the surface of the melanomas made the analysis inaccurate. In addition, overexposed images of SKs are not suitable for this analysis, because their signal level does not fit the auto thresholding, and they have resulted in very low values among the SKs despite their high number of highly autofluorescent particles. Accordingly, the right length of exposure is crucial during the image acquisition.

The novel SK index could differentiate melanomas from SKs with a sensitivity of 91.9% and specificity of 57.0%. This method may harbor a potential to screen melanomas in the everyday practice among general physicians (GPs), as it is capable of differentiating melanomas from benign SKs. Computer-assisted melanoma diagnosis is focusing on the differential diagnosis of melanoma from pigmented lesions, mostly nevi. There are many promising tools and applications, many of which have very high sensitivities and specificities [65], and they often use artificial intelligence based computer-assisted devices (CAD) to differentiate the lesions. According to a meta-analysis of 8 studies, CAD using multispectral images can differentiate melanoma with an average sensitivity of 92.9% and specificity of 43.6%, whose data are comparable with our findings [73].

Similar studies have been carried out using different imaging techniques, including Raman spectroscopy, where it proved to be applicable to differentiate malignant skin lesions from benign lesions, where 147 seborrheic keratoses were in the benign subgroup. This technique had a 99% sensitivity and a specificity of 33.5%, and, both in the initial and the developer group achieved a sensitivity of 90% and specificity of 82.1% until the end of the development phase [74]. Another research proved the feasibility of Raman spectroscopy as a screening device in centers that can differentiate skin tumors in the near-infrared spectra, also ranging with a high positive predictive value from 20% to 52% and a negative predictive value ranging from 73% to 99% [75]. In these studies, Raman spectroscopy proved capable of not only the differentiation melanoma from SK, but also from other malignant tumors, such as basal cell carcinoma and squamous cell carcinoma [74,75]. These results are comparable to our findings with high sensitivities and specificities; however, Raman spectroscopy is expensive and not an easily accessible tool and can be hardly implemented to the everyday practice by a general physician, but it would be an excellent modality in specialized skin cancer centers. LED-based MSI is a more accessible device, which would be suitable in a non-expert practice to screen patients in order to differentiate melanoma from seborrheic keratosis.

We found that melanoma may be differentiated from SK with the use of intensity descriptors and the SK index (1). The subjectiveness of clinical examinations in dermatology is very high, and there is an emerging need of objective quantitative parameters. This imaging algorithm implemented into this cost-effective tool may help the everyday practice of general physicians in the future. We believe that our findings may very well be used for future artificial intelligence analysis.

## Data Availability

The data that support the findings of this study are available from the corresponding author N.K., upon reasonable request.
